# Robust Pose Estimation and Size Classification for Unknown Dump Truck Using Normal Distribution Transform

**DOI:** 10.3390/s25185701

**Published:** 2025-09-12

**Authors:** Kai Imai, Kota Watanabe, Hiroaki Okabe, Takafumi Matsuyama, Atsushi Shirao, Takuma Ito

**Affiliations:** 1 Graduate School of Engineering, The University of Tokyo, 7-3-1 Hongo, Tokyo 113-8656, Japan; 2 Komatsu Ltd., Tsu 23 Futsu-machi, Komatsu-shi 923-0392, Ishikawa, Japan

**Keywords:** dump truck, pose estimation, size classification, normal distribution transform, LiDAR, point cloud, construction automation

## Abstract

Labor shortage has been a severe problem in the Japanese construction industry, and the automation of construction work has been in high demand. One of the needs is the automation of soil loading onto dump trucks. This task requires pose estimation and size classification of the dump trucks to determine the appropriate loading position and volume. At actual construction sites, specifications of dump trucks are not always known in advance. However, most of the existing methods cannot robustly estimate the pose and the size of such unknown dump trucks. To address this issue, we propose a two-stage method that estimates the pose of dump trucks and then classifies their size categories. We use Normal Distribution Transform (NDT) for pose estimation of dump trucks. Specifically, we utilize NDT templates of dump trucks which distinguish global differences among size categories and simultaneously absorb local shape variations within each category. The proposed method is evaluated by data in a real-world environment. The proposed method appropriately estimates the pose of dump trucks under various settings of positions and orientations. In addition, the method correctly classifies the observed dump truck with all three predefined size categories. Furthermore, the computation time is approximately 0.13 s, which is sufficiently short for practical operation. These results indicate that the method will contribute to the automation of soil loading onto dump trucks with unknown specifications.

## 1. Introduction

**Figure 1:**
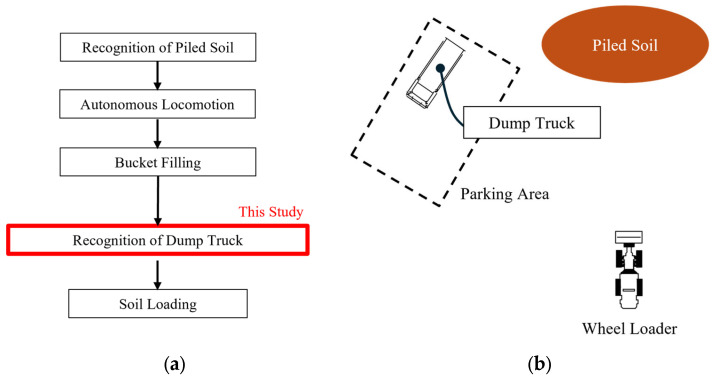
Assumed situation in automated soil loading. (**a**) Component technologies and the pipeline. (**b**) Layout of the wheel loader, dump truck, and soil.

In Japan, the construction industry has faced a labor shortage due to an aging population and declining number of skilled workers. Specifically, the number of construction technicians has been basically decreasing over the past ten years [1]. According to the statistical survey about the working population of the construction industry in 2024 [1], those aged 60 and above account for over 25% of the total, while those under 30 account for about 12%. For this social background, the automation of construction tasks has become necessary. One of the major unsolved issues is to automate soil loading onto dump trucks with wheel loaders. Figure 1a illustrates the component technologies and the pipeline for soil loading. In this case, the automation consists of some processes such as bucket filling [2,3], autonomous locomotion [4,5], and soil loading onto dump trucks [6,7]. Among these processes, we focus on the recognition of dump trucks for soil loading based on point cloud data. To be more precise, the wheel loaders require pose estimation and size classification of the dump trucks to determine the appropriate loading position and capacity. Figure 1b illustrates the assumed layout of the wheel loader, dump truck, and soil. In this setup, the automatic loading system needs to estimate the pose and classify the size of the dump truck located in front of the wheel loader for automatic soil loading.

**Figure 2:**
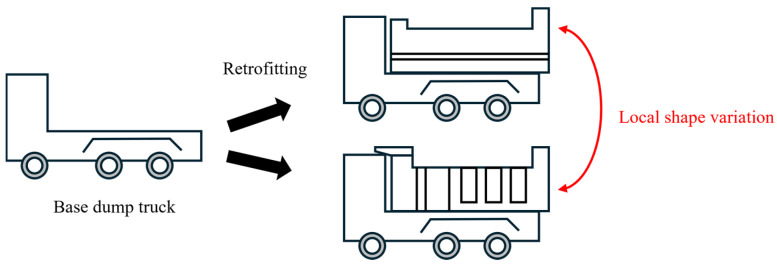
Local shape variations in dump trucks due to vessel design.

One of the key challenges in actual construction sites is that the specifications of dump trucks are not always known in advance. Because dump trucks from various contractors enter and leave actual construction sites, it is difficult for the automatic loading system to fully manage their specifications. This situation raises two issues. First, classification of size categories is required to estimate the loading capacity of each truck. Second, pose estimation becomes more difficult due to the increased variability in truck shape within each size category. As for the second issue, Figure 2 illustrates the local shape variations in the unknown dump trucks due to the design of the vessel. Because various contractors retrofit base vehicles with specific vessels, local shape variations occur even within the same size category depending on the vessel design. Therefore, it is necessary to develop a method that performs both pose estimation and size classification for dump trucks with unknown specifications. To address this challenge, the method must distinguish global differences among size categories in size classification and also absorb local shape variations within each category in pose estimation. In addition, for practical implementation on construction machinery, the method should perform fast under limited computational resources.

Based on the above considerations, this study proposes a two-stage method based on Normal Distribution Transform (NDT) [8], which is a method of point cloud registration. In existing research for recognition of dump trucks using point clouds, truck poses have often been estimated through point cloud registration between an observed point cloud and a reference point cloud. Among such approaches, Iterative Closest Point (ICP) [9] has been widely used [10,11]. ICP matches a pair of point clouds by minimizing the distance between each point in the observed point cloud and its nearest neighbor in the reference point cloud. This approach works well when the specifications of the observed dump truck are known in advance and a corresponding reference point cloud can be prepared. However, as discussed above, such assumptions are not always met in the actual construction sites. In addition, although deep-learning-based methods have also been investigated for dump trucks [12], they still have challenges in terms of the cost of collecting sufficient training data. On the other hand, NDT represents a reference point cloud by a set of normal distributions and minimizes the distance between the observed point cloud and these distributions. Because NDT approximates local shape variations with probability distributions, it is expected to be more robust against shape variations between the observed dump truck and the reference point cloud in the first step. For size classification, NDT alone does not provide a direct solution. Accordingly, we extend it to a parallel comparison based on the fact that NDT optimizes the matching score to determine the transformation. Specifically, we prepare multiple reference point clouds that represent different size categories and conduct NDT-based pose estimations in parallel. At least in Japan, dump truck sizes can be roughly categorized into a limited number of predefined categories, and it is practical to prepare the corresponding reference point clouds. Size classification is then performed by selecting the size category that provides the highest optimized score in the second step.

**Figure 3:**
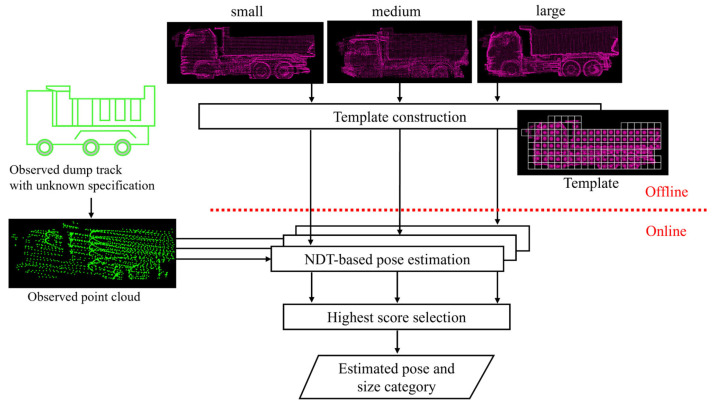
Conceptual diagram of the proposed method.

Figure 3 shows the conceptual diagram of the proposed method. First, an NDT template is constructed for each size category of dump trucks. These templates are constructed to distinguish global differences across size categories while absorbing local shape variations within each size category. Then, the pose estimation is performed by matching the input point cloud with the template of each size category by NDT. After that, the size classification is achieved by comparing the optimized scores across size categories and selecting the one with the highest score. In this way, the proposed method achieves robust pose estimation and size classification for unknown dump trucks.

The main contributions of this study are as follows:

- Proposal of a two-stage method for pose estimation and size classification of dump trucks with unknown specifications using NDT templates;
- Experimental validation of the proposed method using real-world data under various settings of different positions and size categories.

The remainder of this paper is organized as follows: Section 2 reviews the existing research on the recognition of dump trucks using point cloud data. Section 3 explains the details of the proposed method. In Section 4 and Section 5, the proposed method is evaluated by real-world data for pose estimation and size classification, respectively. Section 6 organizes discussion and limitations in this study. Finally, Section 7 presents the conclusions.

## 2. Related Work

This study focuses on technologies of pose estimation and size classification of dump trucks using point cloud data. Although we use point cloud registration in the proposed framework, Section 2.1 reviews other approaches to pose and shape estimation and explains motivation for using point cloud registration. Then, Section 2.2 summarizes existing registration methods and describes the advantage for using NDT. Finally, Section 2.3 reviews recognition methods of dump trucks in construction automation and emphasizes that pose and size estimation for dump trucks with unknown specifications has not been sufficiently investigated.

### 2.1. Pose and Shape Estimation Using Point Cloud

In the broader field of automobile recognition, several methods have been proposed to estimate the pose of automobiles by finding a 2D boundary rectangle that fits best to a point cloud. These methods aim to extract pose information from partially observed point clouds of surrounding vehicles captured by onboard LiDAR, using simplified rectangular representations and iterative optimization. Zhang et al. [13] proposed a fitting method by searching for the optimal orientation angle. Liu et al. [14] proposed a method for generating candidate attitude angles using a convex hull and fitting a rectangle to the point cloud. Baeg et al. [15] developed a method for fitting rectangles that encodes the point cloud into 2D grids and uses the number of points in the cell and the center coordinates. Although such rectangle fitting is fast and robust for partially observed point clouds, it is too simplified and insufficient for accurate pose estimation required in soil loading. In addition to such 2D rectangle fitting approaches, deep learning-based methods for detecting 3D bounding boxes have also advanced. While the former iteratively fits rectangles to the observed point cloud through optimization, the latter is trained on labeled datasets and directly outputs bounding boxes for the observed point cloud. For example, Yan et al. [16] proposed SECOND, which applies sparse convolutions on voxel features. Lang et al. [17] proposed PointPillars, which encodes point clouds into vertical pillars and then processes them with 2D convolutions. Shi et al. [18] proposed PV-RCNN, which integrates voxel-based features and keypoint-based features in a two-stage framework. While these methods have high detection accuracy, 3D box representations remain a simplified approximation that does not fully capture the detailed geometry of automobiles and are insufficient for soil loading. To be more precise, automation tasks such as soil loading require recognizing not only the whole dump truck but also more detailed parts, such as the vessel. In contrast, because point cloud registration matches the observed point cloud with a reference point cloud or template, it helps to recognize more detailed parts of the dump truck.

Apart from the above simplified rectangle or box detection approaches, some studies have aimed to recognize more detailed automobile size and geometry. Zhang et al. [19] classified automobiles using aerial LiDAR data based on differences in height and overall shape. Kraemer et al. [20] developed a method that estimates vehicle shape using polylines constrained by free-space information. In addition, Kraemer et al. [21] employed multi-layer laser scanners and used ICP-based registration to estimate both motion and shape through point cloud accumulation. Monica et al. [22] introduced a recurrent neural network-based approach that estimates shape and pose from sequential LiDAR data. Other approaches, such as that of Ding et al. [23], use multiple monocular cameras and convolutional neural networks to detect vehicle key points and estimate 3D geometry. Monica et al. [24] further extended shape estimation by converting stereo depth maps into point clouds using Pseudo-LiDAR++. While they achieve detailed shape reconstruction using various representations, such as polylines, keypoints, and accumulated point clouds, they typically assume dense observations of the vehicle. In contrast, in the assumed setup in this study, only a partial point cloud of a dump truck is observed by LiDAR. Moreover, computational efficiency is critical for practical operation.

In summary, methods that detect vehicles as 2D rectangles or 3D bounding boxes are too simplified, while those that aim at more detailed shape estimation have high computational cost. In contrast, because this study focuses on dump trucks representative templates can be prepared in advance. Therefore, the proposed method employs point cloud registration to match the observed point cloud with such templates. To be more precise, the proposed method performs NDT-based pose estimation with multiple normal distribution templates that represent different dump truck size categories in parallel. By comparing the NDT-based scores, our method classifies the size of the dump truck in a computationally efficient way.

### 2.2. Methods of Point Cloud Registration

As described in Section 2.1, point cloud registration is used for pose estimation of dump trucks in this study. Point cloud registration methods estimate the pose by searching for the coordinate transformation that matches the observed point cloud with a reference point cloud. Among them, one of the most representative and traditional approaches is ICP. ICP matches an observed point cloud with a reference point cloud by iteratively searching for the nearest neighbor points [9]. On the other hand, in NDT, the reference point cloud is divided into voxels at uniform intervals, and the distribution of the reference point cloud within each voxel is modeled as a normal distribution. Matching is then performed by minimizing the Mahalanobis’ distance between each observed point cloud after transformation and the corresponding normal distribution [8]. Magnusson et al. [25] compared the performance of ICP and NDT in terms of robustness to initial pose and computation time. In their experiments, NDT converges from a larger range of initial poses and performs faster than ICP. In addition, an extension of ICP called Generalized-ICP (GICP) [26] has been proposed. In GICP, each point in the reference and observed point cloud is assumed to be generated from a different normal distribution. Although GICP achieved higher accuracy than both ICP and NDT, it requires more computation time than NDT.

In the assumed setup in this study, the specifications of the observed dump trucks are unknown, and it is impossible to prepare a reference point cloud of the same vehicle. Therefore, local shape variations within the same size category become a challenge in the case of ICP, which minimizes the distance between individual points. In contrast, GICP and NDT can handle this issue by modeling the reference point cloud not as discrete points but as spatial distributions. Due to its computational efficiency, this study employs NDT and utilizes it for both pose estimation and size classification.

In addition to the above traditional methods, several learning-based registration methods have also been proposed with the development of deep learning. Aoki et al. [27] proposed PointNetLK, which integrates PointNet [28] with a modified Lucas–Kanade algorithm for point cloud registration. Wang and Solomon [29] proposed Deep Closest Point, which employs PointNet or DGCNN [30] to extract point features and applies attention-based soft matching to estimate rigid transformations. Although these deep-learning-based methods achieve higher accuracy compared with traditional methods, they also have a challenge in training. Collecting sufficient labeled data requires considerable cost, and their generalization for out-of-domain data, such as point clouds captured by different LiDAR sensors, is limited. In contrast, NDT template can be constructed if one representative dump truck point cloud is available for each size category. This makes the implementation more practical.

### 2.3. Recognition Methods of Dump Trucks in Construction Automation

To determine the appropriate loading position, the automatic loading system requires pose estimation of the dump truck. One possible approach is to equip the dump truck with sensors or markers in advance and then utilize this information for pose estimation [31,32]. However, as mentioned earlier, because dump trucks from various contractors operate in actual construction sites, such information is not always available. Apart from this approach, various studies have investigated methods based on point cloud data. Stentz et al. [33] estimated the truck pose by fitting planar regions extracted from point cloud to a six-plane dump truck model. Phillips et al. [34] generated multiple pose hypotheses of a dump truck in advance and then performed pose estimation by evaluating their likelihood against the acquired point cloud data. Lee et al. [10] proposed a two-stage pose estimation method which consists of an initial transformation using a hexahedral truck model and a refinement using ICP. Sugasawa et al. [11] estimated the poses of dump trucks with LiDAR mounted on an excavator using ICP. An et al. [12] developed a deep point cloud registration network aimed at fusing local and global features and applied it to pose estimation of a dump truck. All of these methods estimate poses by preparing template model or reference point cloud in advance and matching the observed point cloud to it. As a result, the vessel area of the dump truck can be identified and utilized for following soil loading.

However, the above methods have assumed that the dump truck’s specifications are known in advance. Therefore, these methods are not robust to the local shape variation in actual construction sites, where the specifications of the dump trucks are not always known in advance. Although learning-based registration approaches may be able to address the issue of shape variation, their applicability is limited by the cost of data collection and the computational resources. To overcome these limitations, we utilize an NDT-based pose estimation method. The important point is that NDT represents reference dump trucks as normal distribution templates, which absorb local shape variations in the dump trucks and perform sufficiently fast for practical operation.

## 3. Method

### 3.1. Overview

**Figure 4:**
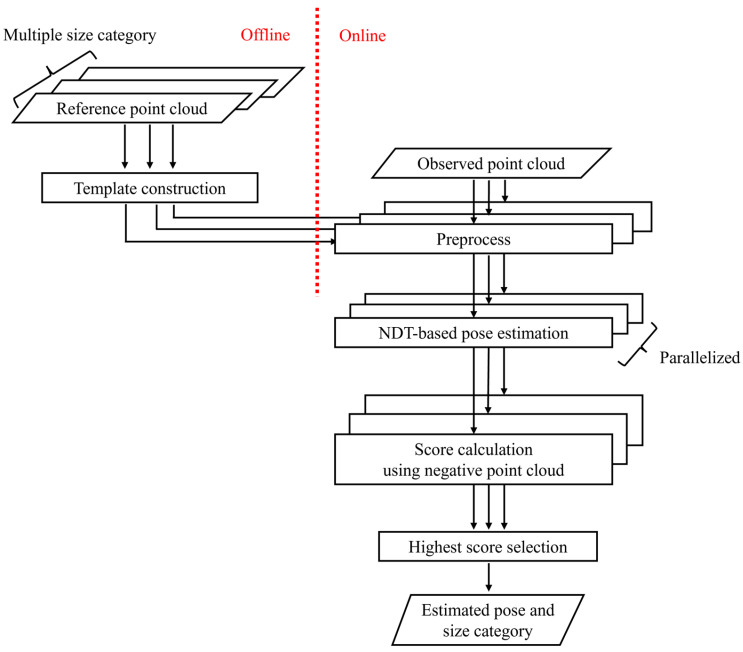
Pipeline of proposed method. Before online operation, template construction is performed in advance (Section 3.2). Online operation consists of preprocess (Section 3.3), NDT-based pose estimation (Section 3.4), and score calculation (Section 3.5).

Figure 4 shows the pipeline of the proposed method. In advance, a set of templates is constructed from reference point clouds for each size category (Section 3.2). During online operation, first, the point cloud of the dump truck is observed by the LiDAR sensors mounted on the wheel loader. The observed point cloud is then preprocessed with a rectangle fitting method to obtain an initial transformation for the following NDT process (Section 3.3). Next, NDT iteratively updates transformation parameters by matching the observed point cloud with pre-constructed templates (Section 3.4). These preprocess and NDT-based pose estimation are performed in parallel for multiple templates. After the pose estimation for each size category, the method compares the scores which are designed for size classification and then selects the one with the highest value (Section 3.5). To be more precise, we introduce negative point clouds into the conventional NDT score to correctly distinguish between different size categories.

In this study, pose estimation is formulated as the problem of estimating a coordinate transformation that matches the observed point cloud with a template whose pose is already known. Because both the wheel loader and the dump truck are positioned horizontally on flat ground at the construction site, we consider the 2-D transformation parameter p defined as follows in this study:

(1) $$p=(t_{x},\;t_{y},\;\phi_{z})$$

where $t_{x}$ and $t_{y}$ represent translational parameters along the *x*-axis and *y*-axis, respectively, and $\phi_{z}$ denotes the yaw angle. When a point ($x_{j}$, $y_{j}$, $z_{j}$) in the observed point cloud is transformed by this transformation parameter p, the position of the point after the transformation ($x_{j}\prime$, $y_{j}\prime$, $z_{j}\prime$) is expressed as follows:

(2) $$\left(\begin{array}{c} x_{j}\prime \\ y_{j}\prime \\ \;z_{j}\prime \end{array}\right)=\left[\begin{array}{ccc} cos\phi_{z} & -sin\phi_{z} & 0\\ sin\phi_{z} & cos\phi_{z} & 0\\ 0 & 0 & 1 \end{array}\right]\left(\begin{array}{c} x_{j}-x_{0}\\ y_{j}-y_{0}\\ z_{j}-z_{0} \end{array}\right)+\left(\begin{array}{c} x_{0}\\ y_{0}\\ z_{0} \end{array}\right)+\left(\begin{array}{c} t_{x}\\ t_{y}\\ 0 \end{array}\right)$$

where $\left(x_{0},y_{0},\;z_{0}\right)$ indicates the center of the template. In other words, the transformation is defined as a translation in the xy-plane and a rotation around the *z*-axis that passes through the center of the template.

### 3.2. Construction of Normal Distribution Template

Before the online operation, normal distribution templates are constructed for different size categories using reference point clouds. These reference point clouds represent the typical shape of dump trucks in each category, and they are prepared in advance. Specifically, they are created by merging point clouds of a dump truck captured from all directions. We emphasize that the dump trucks which the reference point clouds represent are not always identical to those which will be observed during actual operation. Local shape variations may exist between the reference and observed dump trucks, even within the same size category.

The following part describes the process of constructing a single template from a reference point cloud. First, the reference point cloud is divided into a grid of voxels. Then, the mean vector $\mu_{k}$ and covariance matrix $\Sigma_{k}$ are computed for a subset of points within the voxel k as follows:

(3) $$\mu_{k}=\frac{1}{n_{k}}\sum_{i=1}^{n_{k}}x_{k,i}$$

(4) $$\Sigma_{k}=\frac{1}{n_{k}}\sum_{i=1}^{n_{k}}\left(x_{k,i}-\mu_{k}\right){\left(x_{k,i}-\mu_{k}\right)}^{T}$$

where $n_{k}$ represents the number of points within voxel k, and $x_{k,i}$ ($i=1,\ldots ,n_{k}$) represents the i-th point within the voxel k.

**Figure 5:**
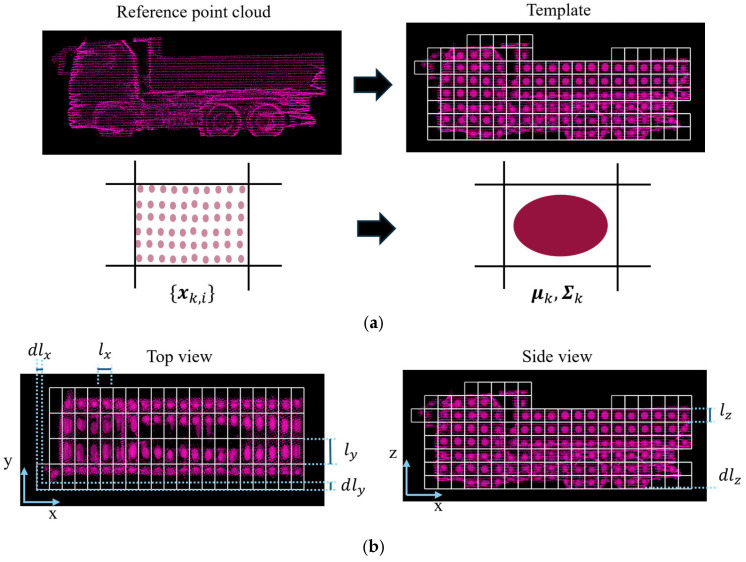
Visualization of Template. In this case, ($l_{x}$, $l_{y}$, $l_{z}$) is (0.4, 0.8, 0.4) [m] and (${dl}_{x}$, ${dl}_{y}$, ${dl}_{z}$) is (0.2, 0.2, 0.0) [m]. (**a**) Reference point cloud and constructed template. (**b**) Top view and side view of the template with associated parameters.

Figure 5a illustrates the reference point cloud and constructed template. As shown in Figure 5a, an ellipsoid represents the 95% confidence region of the normal distribution in each voxel. In addition, Figure 5b illustrates how the template is divided into a voxel grid. The voxel grid is defined by 6 parameters: The voxel sizes ($l_{x}$, $l_{y}$, $l_{z}$) and the offsets (${dl}_{x}$, ${dl}_{y}$, ${dl}_{z}$). As an example, the process of the voxel placement along the *x*-axis is described as follows:

1. The point with the smallest x-coordinate is extracted from the reference point cloud.
2. The starting line of the grid is then determined by subtracting the offset ${dl}_{x}$ from the x-coordinate of the above extracted point.
3. From this starting point, the space is divided along the *x*-axis at intervals *l*_x_

The same process is applied to the *y*-axis and *z*-axis using voxel sizes $l_{y}$ and $l_{z}$, and offsets ${dl}_{y}$ and ${dl}_{z}$, respectively.

### 3.3. Preprocess

To avoid convergence to an unfavorable local optimum in the NDT-based pose estimation, a preprocess is conducted to roughly estimate the truck’s pose before the NDT. Although applications that deal with large-scale point clouds often require downsampling or compression [35,36] to improve data transfer speed or execution efficiency, this study uses only a single frame and does not include such processing in the preprocess. This preprocess consists of the following two steps:

1. Filtering of the observed point cloud based on the predefined parking area and height threshold.
2. Rectangle fitting to approximate the truck’s shape with a rectangle for estimation of an initial transformation.

The details of each step are explained in the following parts.

#### 3.3.1. Filtering Based on Parking Area and Height

First, the observed point cloud is filtered based on the positions. Specifically, points outside the predetermined parking area are removed. In addition, to remove noise and ground points, only points above a certain height threshold are retained. This process ensures that the filtered point cloud primarily represents the dump truck.

#### 3.3.2. Initial Transformation Using Rectangle Fitting

**Figure 6:**
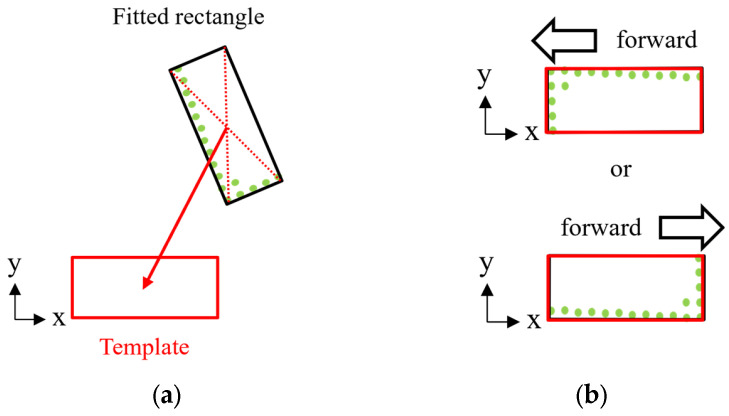
Process of pre-transformation using rectangle fitting. Green dots indicate filtered point cloud, and red dotted lines represent diagonals of fitted rectangle. (**a**) Translational transformation. (**b**) Ambiguity of forward-backward orientation estimation.

After the filtering, the rectangle fitting method [13] is applied to the filtered point cloud to obtain a 2-D bounding rectangle. This rectangle approximates the horizontal projection of the filtered points. Figure 6 illustrates the process for deriving the initial transformation. Here, the coordinate system is defined such that the long side of the template is aligned with the *x*-axis. First, the center of the bounding rectangle is computed from its four vertices. A translational transformation is then applied to align this center with the center of the template, as shown in Figure 6a. Next, a rotational transformation is performed to align the orientation of the bounding rectangle with that of the template. However, because this initial transformation is based only on the geometric shape of the rectangle, it cannot distinguish between forward and backward orientations of the dump truck, as shown in Figure 6b. To address this ambiguity, two types of initial transformations are used in the following NDT-based pose estimation. One transformation is a 180-degree rotation of the other. In other words, one corresponds to the correct orientation and the other to the reversed orientation.

### 3.4. NDT-Based Pose Estimation

In this study, we assume that the yaw angle is estimated with sufficiently high accuracy in the preprocess, and only the translational transformation parameters are updated in the NDT-based pose estimation for computational cost. First, the observed point cloud is transformed by a parameter p according to Equation (2). The probability density function is evaluated for all transformed points $x_{j}\;(j=1,\ldots ,N)$ and all voxels in the template. Here, N denotes the total number of the points in the point cloud. For each point $x_{j}$, let $k_{j}$ denote the nearest voxel in the template. Then, the likelihood of $x_{j}$ is expressed as follows:

(5) $$\left(x_{j}\right)=\frac{1}{{\left(\sqrt{2\pi }\right)}^{n}\sqrt{\left|\Sigma_{k_{j}}\right|}}\exp\left(-\frac{{\left(x_{j}-\;\mu_{k_{j}}\right)}^{T}{\Sigma_{k_{j}}}^{-1}\left(x_{j}-\;\mu_{k_{j}}\right)}{2}\right)$$

where $\mu_{k_{j}}$ and $\Sigma_{k_{j}}$ denote the mean vector and covariance matrix of a voxel $k_{j}$, and n denotes the dimension of $x_{j}$, which is 3 in this study.

This study employs the NDT formulation proposed by Biber et al. [37], who introduced a mixture model of a normal distribution and a uniform distribution. By incorporating this mixture model and applying further approximations, their method improves both robustness and computational efficiency. Following their formulation, the evaluation function for parameter p becomes computable as a sum of exponential functions [38] as follows:

(6) $$E\left(p\right)=\frac{1}{N}\sum_{j=1}^{N}d_{1}\exp\left(-d_{2}\frac{{\left(x_{j}-\mu_{k_{j}}\;\right)}^{T}{\Sigma_{k_{j}}}^{-1}\left(x_{j}-\mu_{k_{j}}\;\right)}{2}\right)$$

where $d_{1}$ and $d_{2}$ are constraints.

To optimize $E\left(p\right)$, the Newton method has been generally used in existing research. However, in this study, we employ the Euler method instead. This decision is based on two reasons. First, we found that the Newton method exhibits unstable behavior near inflection points, which can lead to divergence in the update of transformation parameters. Second, the Newton method requires the computation of the Hessian matrix, resulting in a high computational cost. Based on these considerations, we employed the Euler method to improve stability and efficiency in NDT-based pose estimation for dump trucks.

In the Euler method, the transformation parameter’s increment ∆p is given as follows:

(7) ∆p=h·g

where h represents the step size and g is the gradient vector of $E\left(p\right)$. Because the preprocess provides a rough initial transformation and the NDT aims to refine this transformation, we limit the maximum value of ∆p by setting h as follows:

(8) $$h=\left\{\begin{array}{c} \;\;\;1\;\;\;\;\left(if\;\left|g\right|\leq 0.01\right)\\ \frac{0.01}{\left|g\right|}\;\left(if\;\left|g\right|>0.01\right) \end{array}\right.$$

This ensures a maximum movement of 0.01 m within one iteration.

As described in Section 3.1, the preprocess and the NDT-based pose estimation are performed with multiple templates corresponding to different size categories. In addition, as described in Section 3.3, to determine the correct forward-backward orientation, two different initial transformations are inputted to the NDT-based pose estimation. Therefore, for the observed point cloud, the system conducts NDT-based pose estimation in parallel for all combinations of size categories and forward-backward orientations. In other words, if S represents the number of size categories, the system performs 2S NDT-based evaluations in parallel, considering both possible orientations for each category.

### 3.5. Size Classification with Negative Point Cloud

For the 2S NDT-based pose estimations which are conducted in parallel, the system performs a two-step score selection. First, for each size category, it compares the two NDT scores with different initial orientations and selects the higher score. This first step determines the correct forward-backward orientation within each size category. Next, among the S NDT results that correspond to different size categories, the system selects the highest score. This second step determines the correct size category. Through this two-step process, the system determines both the forward-backward orientation and the size category of the observed dump truck.

**Figure 7:**
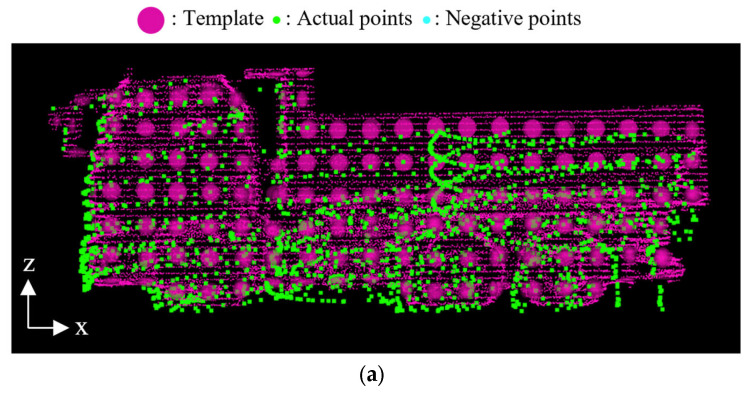

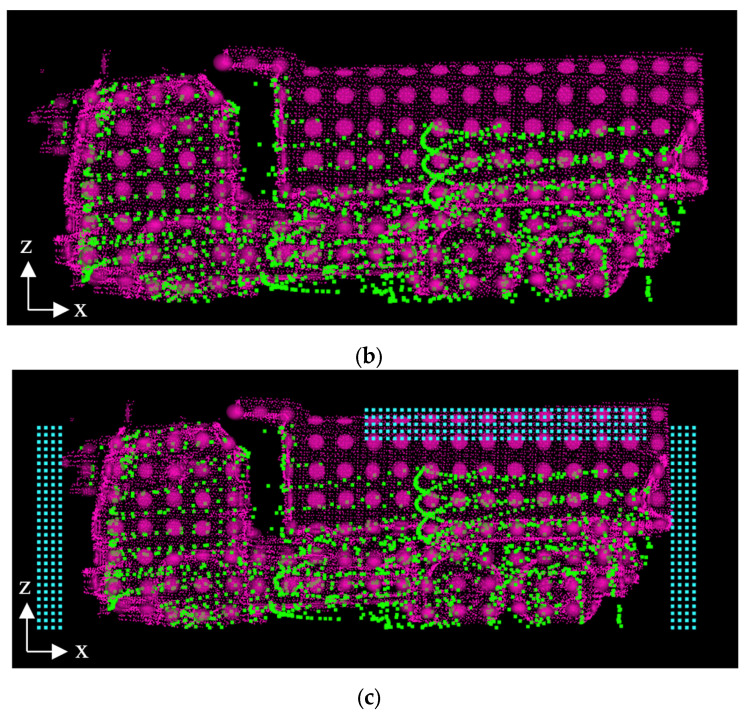
Template and actual point cloud when an invalid higher score situation. (**a**) valid overlap between observed point cloud and template. (**b**) invalid overlap between observed point cloud and template. (**c**) Negative point cloud incorporated into (**b**).

However, in the second step of the score selection, the size classification cannot be reliably performed only by comparing the conventional NDT scores. Because NDT maximizes the overlap between the template and the transformed point cloud, it may result in an invalid higher score when the template corresponds to a larger dump truck than the observed point cloud. Figure 7a,b illustrate valid and invalid overlap between the point cloud and the template, respectively. In the figures, the green points represent the observed point cloud, and a set of red ellipsoids represents the template. To mitigate the issue of this invalid overlap, we propose a method to reduce the score when the template size is larger than the actual dump truck. Specifically, in the second step of the score selection, we introduce a virtual “negative” point cloud around the observed point cloud, which overlaps with the larger templates as shown in Figure 7c. In the figure, the blue points represent the negative point cloud. This negative point cloud contributes as a negative term when calculating the NDT score. The NDT score incorporating the negative point cloud is expressed as

(9) $$score=\frac{1}{N}\sum_{j=1}^{N+M}\omega_{j}d_{1}\exp\left(-d_{2}\frac{{\left(x_{j}-\mu_{k_{j}}\right)}^{T}\Sigma_{k_{j}}^{-1}\left(x_{j}-\mu_{k_{j}}\right)}{2}\right)$$

where N and M represent the number of points of the actual and negative point cloud, respectively. Here, the negative term is incorporated into the score by setting $\omega_{j}$ as follows:

(10) $$\omega_{j}=\left\{\begin{array}{c} 1\;\left(if\;x_{j}\;belongs\;to\;actual\;point\;cloud\right)\\ -1\;\left(if\;x_{j}\;belongs\;to\;negative\;point\;cloud\right) \end{array}\right.$$

**Figure 8:**
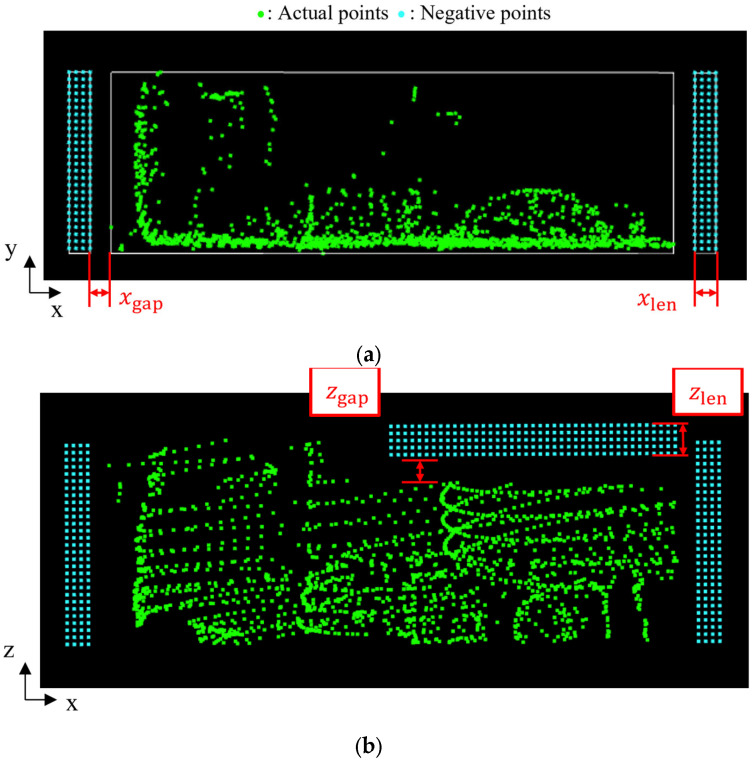
Visualization of transformed point cloud and negative point cloud. White lines around green points represent fitted rectangle, and white lines around blue points represent assignment area for negative points. (**a**) Top view. (**b**) Side view.

Figure 8 illustrates the positioning of the negative point cloud. In the figure, the green points represent the observed point cloud, while the blue points represent the negative point cloud. The negative point cloud is assigned to the areas in front of and behind the observed point cloud, as well as above the vessel. The spatial range of the negative point cloud in front of and behind the dump truck is defined by the fitted rectangle obtained in the preprocess and parameters $x_{gap}$ and $x_{len}$, as shown in Figure 8a. Here, $x_{gap}$ represents the gap distance between the negative point cloud and the fitted rectangle. Moreover, $x_{len}$ represents the length of the negative point cloud range. In addition, the range in the y-direction is defined by the minimum and maximum y-coordinates of the fitted rectangle. Similarly, the range of the negative point cloud above the vessel is defined by $z_{gap}$ and $z_{len}$, as shown in Figure 8b. In addition, the range in the x-direction is defined by the x-coordinate of the center of the fitted rectangle and the maximum x-coordinate. Furthermore, the interval between the negative points $d_{neg}$ is also a parameter in the assignment process. These five parameters $x_{gap}$, $x_{len}$, $z_{gap}$, $z_{len}$, and $d_{neg}$ are designed based on the requirements for size classification, and their numerical values are described in Section 5.1.

## 4. Evaluation of Pose Estimation

### 4.1. Experimental Setup

**Figure 9:**
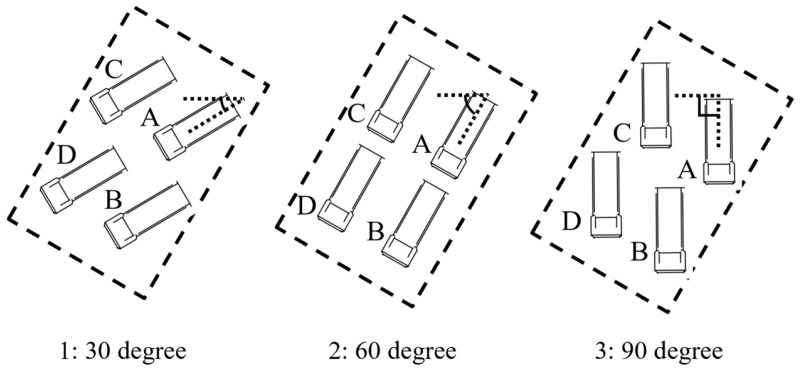
Position and angle conditions of observed data. A–D represent four different dump truck positions.

This section evaluates the performance of the NDT-based pose estimation. To isolate the evaluation of pose estimation from that of size classification, the experiments used a single template that belongs to the same size class as the observed dump truck. It should be noted that the template and the observed dump truck were not identical, and local shape variations existed between them. For this evaluation, point cloud data of a dump truck were collected under 12 different settings. Specifically, the dump truck was parked at four different positions for each of the three orientations. These settings are labeled 1-A to 3-D, as shown in Figure 9. The purpose of this experiment is to evaluate the method’s ability to estimate poses under two types of variation: (1) local shape differences between the template and the observed dump truck within the same size category, and (2) variations in the truck’s position and orientation within the parking area.

The whole process was conducted on a laptop computer with an AMD (Santa Clara, CA, USA)^®^ Ryzen 7 7730U [39] processor on C++ nodes implemented in ROS Noetic. In addition, the data were acquired using two Livox HAP LiDARs mounted on the front of the wheel loader. The specifications of the LiDAR sensor are summarized in Table 1 [40]. In addition, the maximum number of NDT update iterations was set to 20.

### 4.2. Evaluation Method

The evaluation is conducted from three aspects: (1) correctness of forward-backward orientation, (2) error in the estimated yaw angle $\phi_{z}$, and (3) errors in the estimated translational parameters $t_{x}$ and $t_{y}$. Each aspect is described below.

#### 4.2.1. Forward-Backward Orientation

As described in Section 3.3, the proposed method performs pose estimation for both forward and backward orientations of the dump truck and selects the one with the higher NDT score. The orientation is considered correct if the selected result matches the actual orientation of the observed dump truck.

#### 4.2.2. Error in Yaw Angle $\phi_{z}$

To evaluate the rotational accuracy, two points are manually selected in advance, and the line connecting these two points is used as a landmark line. Specifically, the landmark lines are selected on the side surface of the vessel for both the reference and observed point clouds. After applying the estimated transformation, the yaw angle error is calculated as the difference between the slopes of the reference line and the transformed line.

#### 4.2.3. Error in Translational Parameters $t_{x}$ and $t_{y}$

To evaluate translational accuracy, a feature point is manually selected as a landmark in advance. In this study, the left mirror of the dump truck is selected as the landmark point. To cancel out the rotational error, the observed point cloud is first rotated by the error in $\phi_{z}$ after pose estimation. Then, the corresponding point in the observed point cloud after transformation is compared with the position in the reference point cloud. The errors in $t_{x}$ and $t_{y}$ are computed as the differences between these two positions.

### 4.3. Results of Single Setting

**Figure 10:**
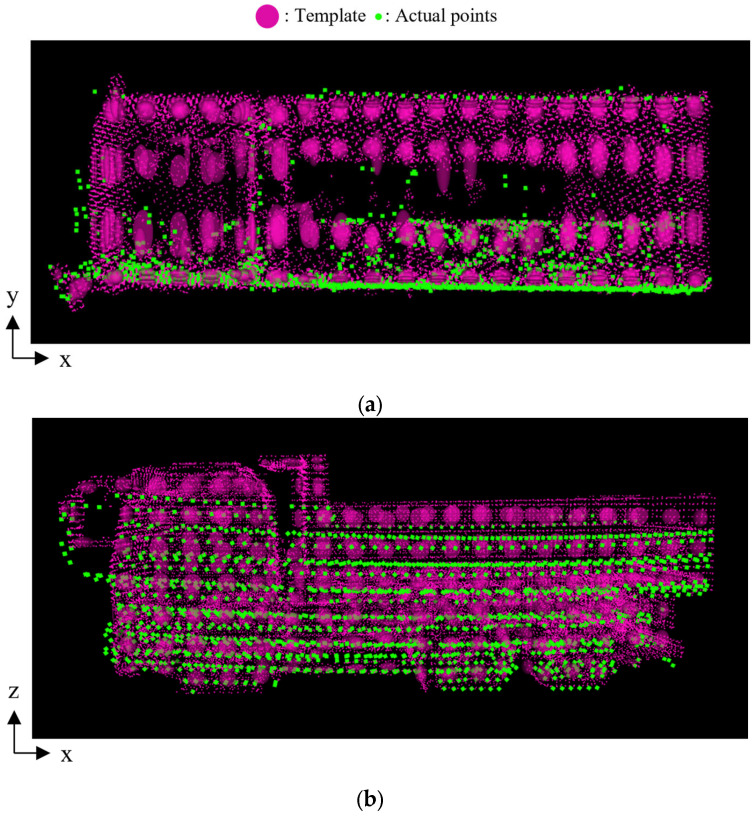
Template and observed point cloud after transformation in case 1-A. (**a**) Top view. (**b**) Side view.

First, we summarize the results of case 1-A, which is shown in Figure 9, for detailed analysis of one setting. Figure 10 shows the observed point cloud after transformation by the NDT-based pose estimation. Specifically, Figure 10a,b show the template and the observed point cloud after transformation in the top and side views, respectively. In the figures, a set of red ellipsoids represents the template, and green points represent the observed point cloud. In this case, the forward-backward orientation is correctly estimated, and the errors in $t_{x}$, $t_{y}$, and $\phi_{z}$ are 0.01 m, 0.10 m, and −0.012 rad, respectively.

### 4.4. Results of All Settings

Next, we analyze the results of all settings for comprehensive performance. Table 2 summarizes the pose estimation results for the 12 settings. In the table, the “Orientation” column indicates whether the forward-backward orientation was correctly estimated. Here, “✓” denotes a correct estimation, while “×” denotes an incorrect estimation. The “Score” column indicates the maximum NDT score. In addition, “Error $t_{x}$”, “Error $t_{y}$”, and “Error $\phi_{z}$” columns indicate the errors in $t_{x}$, $t_{y}$, and $\phi_{z}$, respectively. These estimation errors are evaluated for all rows except for the rows of cases 2-D and 3-C, where the orientation was incorrectly estimated.

**Figure 11:**
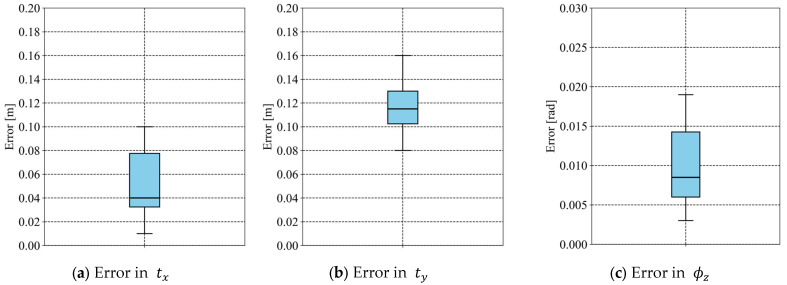
Box plot of absolute errors in (**a**) $t_{x}$, (**b**) $t_{y}$, and (**c**) $\phi_{z}$. Blue area indicates interquartile range.

Figure 11 shows box plots of absolute errors for the ten cases where the orientation was correctly estimated. In these cases, the maximum absolute errors were 0.10 m in $t_{x}$, 0.16 m in $t_{y}$, and 0.019 rad (approximately 1.089°) in $\phi_{z}$. These results indicate that the proposed method can estimate the pose with reasonable accuracy when the correct orientation is estimated.

**Figure 12:**
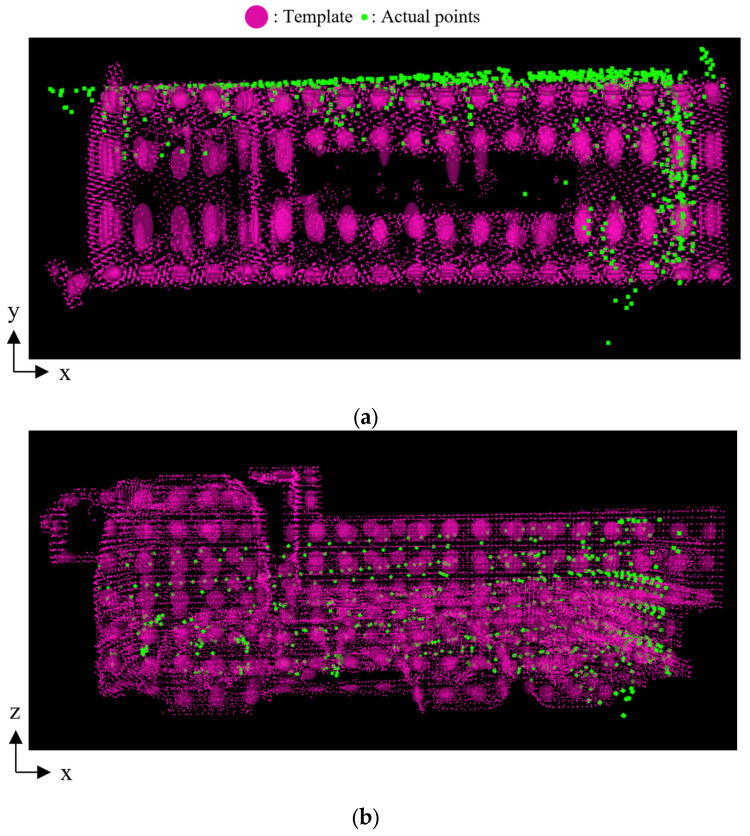
Template and transformed point cloud in case 3-C. (**a**) Top view. (**b**) Side view.

**Figure 13:**
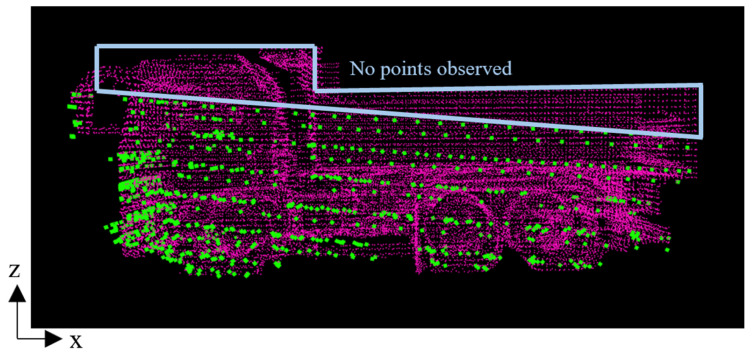
Observed point cloud and reference point cloud in case 3-C. Green points represent observed point cloud, and red points represent reference point cloud.

As an example case of incorrect orientation estimation, Figure 12 shows the result for case 3-C. In this case, the method failed to estimate the correct forward-backward orientation of the dump truck. Figure 13 illustrates a side view of the transformed point cloud overlaid with the reference point cloud. It shows that the top part of the dump truck was outside the LiDAR’s observable area due to the positional relationship between the sensor and the truck. This limitation in observation might contribute to the incorrect pose estimation. Such an incorrect case is primarily due to limitations in sensor coverage rather than the method.

In summary, although the results show certain limitations in cases where the LiDAR observation is insufficient, the proposed method can estimate the pose with reasonable accuracy even under variations in the truck’s position and orientation within the parking area.

## 5. Evaluation of Size Classification

### 5.1. Size Categories of Dump Trucks

**Figure 14:**
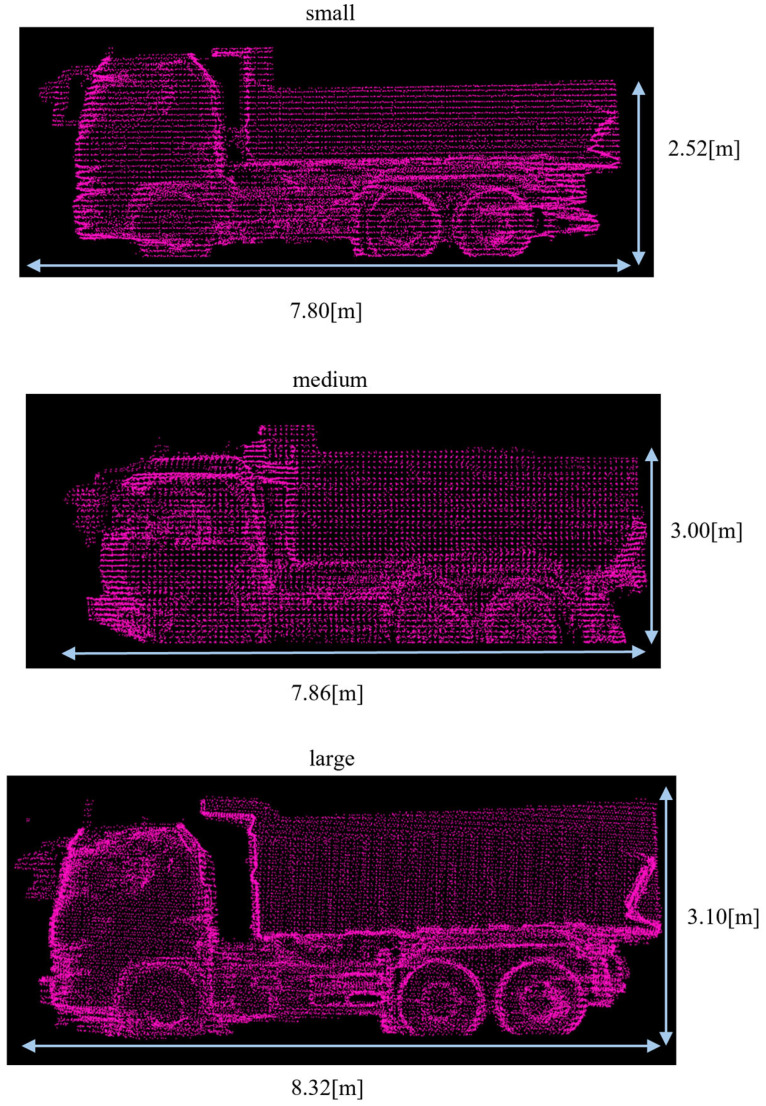
The reference point clouds of small, medium, and large categories. Red points represent reference point clouds.

In this section, we evaluate the size classification performance using multiple templates representing different dump truck size categories. Table 3 summarizes the four dump trucks used as observed point clouds, which are categorized into small, medium, and large. For each size category, one template was constructed using a representative truck; Small A truck was used for small template, Medium for medium, and Large for large. Figure 14 shows the reference point clouds used to construct these templates. To evaluate classification performance, we examined all combinations of the four observed point clouds and the three templates. This setting allows us to evaluate the method’s ability to distinguish between size categories, as well as its robustness to local shape variations within the same category.

As described in Section 3.5, the parameters for placing negative point clouds are determined according to the size specifications of dump trucks. In this evaluation, the parameters were set for classification based on both truck length and vessel height. Specifically, $x_{gap}$ and $x_{len}$ were both set to 0.3 m to classify by dump truck length. Also, $z_{gap}$ and $z_{len}$ were set to 0.4 m and 0.5 m to classify by vessel height, respectively. In addition, the interval between points $d_{neg}$ was set to 0.1 m.

### 5.2. Results

Table 4 shows the results of the size classification before incorporating negative point clouds. The table displays the score computed for each combination of template and observed point clouds. The highest score for each observed point cloud is highlighted in bold and marked with an asterisk. The rightmost column indicates whether the classification was correct. These results show that when a small dump truck was observed, it was frequently misclassified as a larger size. This misclassification occurred because the computed score was incorrectly high when the size category of the template was larger than the observed point cloud. Without negative point clouds, the computed score lacked sufficient penalization for incorrectly overlapped regions between the observed point cloud and the template.

**Figure 15:**
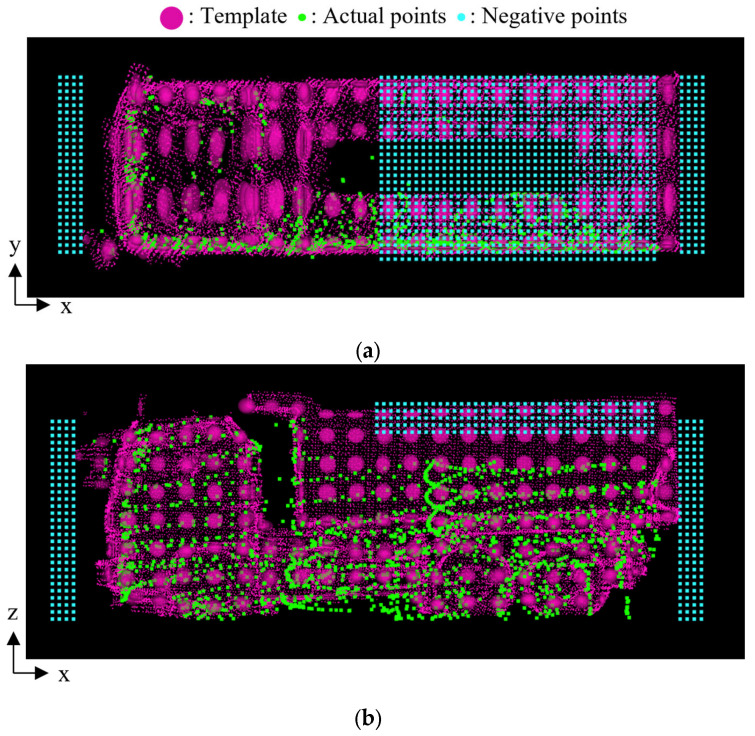
Template, actual point cloud, and negative point cloud in case where dump truck is Small A while the template is Large. (**a**) Top view. (**b**) Side view.

**Figure 16:**
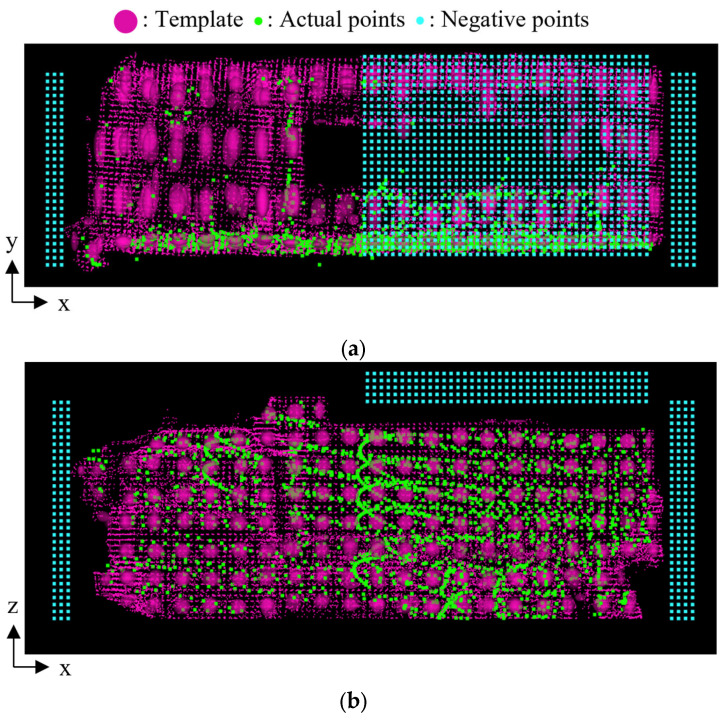
Template, actual point cloud, and negative point cloud in case where both the dump truck and template are Medium. (**a**) Top view. (**b**) Side view.

In contrast, Table 5 shows the results after incorporating negative point clouds. The results demonstrate that appropriate size classification was achieved, even for observed dump trucks belonging to the small category. Figure 15 illustrates the spatial relationship between the template and the observed point cloud after incorporating a negative point cloud. Specifically, the figure shows the combination of the large-size template and the point cloud of Small A truck. In the figure, a set of red ellipsoids indicates the template, the green points indicate the observed point cloud after transformation, and the blue points indicate the negative point cloud. In this case, the score decreases where the template overlaps with the negative point cloud. As a result, the score was reduced from 1.02 to 0.52 in this case. On the other hand, Figure 16 shows the combination of the medium-size template and the observed point cloud of Medium truck. In this case, the score decreased slightly from 1.34 to 1.33 because the template and negative point cloud are far away, as shown in the figure.

These results demonstrate that incorporating negative point clouds into the conventional NDT score enables the system to distinguish global differences between size categories. Consequently, the proposed method can achieve size classification even with the presence of local shape variations within the same category.

Finally, we discuss the computational time of the proposed method. As described in Section 3, when an observed point cloud of a dump truck is input, a total of S pipelines are performed in parallel. In this study, the number of size categories S is 3. Table 6 shows the computation time of each process when the observed point cloud corresponds to the Small A dump truck. The table shows the computation time for each process: Preprocess (Section 3.3), NDT-based pose estimation (Section 3.4), Size classification (Section 3.5), and Total. In this case, even the slowest pipeline, which is the bottleneck of the whole method, required 0.132 s in total. This result demonstrates that the proposed method is suitable for practical operation.

## 6. Discussion and Limitations

This study proposed an NDT-based two-stage framework that addresses pose estimation and size classification of dump trucks with unknown specifications. The method utilizes the probabilistic representation of point clouds to handle local shape variations and extends NDT to parallel comparisons across multiple templates for size classification. The experimental results demonstrated that the method could estimate truck poses with sufficient accuracy while correctly classifying trucks into three predefined size categories. The computational time is approximately 0.13 s per estimation, which is suitable for practical operation under limited computational resources. These results support the feasibility of applying the proposed framework to construction machinery in practice.

Compared with the design approaches in existing studies, two main approaches have been explored for pose estimation of dump truck using point cloud registration: ICP-based methods and deep-learning-based methods. The former generally assumes that the observed dump truck and the reference point cloud correspond to the same vehicle. In contrast, this study verified that pose estimation can be achieved even when the observed dump truck and the template are from different vehicles. On the other hand, although deep-learning-based approaches have the potential to achieve high accuracy and robustness, they require large training data, which have a considerable cost in terms of data collection, and they also have challenges for practical operation on construction machinery with limited computational resources. In contrast, the proposed method only requires representative reference point clouds as templates, which is more practical for construction machinery. Furthermore, by extending NDT to parallel comparisons, the proposed method also achieves size classification of dump trucks. To the best of our knowledge, this function has not been addressed in existing studies.

Despite these contributions, several limitations remain. First, the experiments were limited to three size categories, which does not fully reflect the diversity of actual size categories of dump truck. Although the number of size categories for dump trucks is finite and few, the three categories considered in the experiments are somewhat fewer than those in practice. Therefore, further validation with additional size categories is necessary. Second, the robustness of the method under adverse environmental conditions, such as rain, fog, or dust, was not evaluated. Accordingly, further experiments under real or simulated adverse environments are needed. Third, the proposed method depends on the coverage of LiDAR sensors, and pose errors may occur if parts of the truck lie outside the coverage. Although this issue could be mitigated by carefully selecting the mounting position of LiDAR sensors, in actual application, the sensor placement is determined not only for dump truck recognition but also in consideration of other functions, such as recognition of piled soil or autonomous locomotion. Specifically, one possible way to reduce the problem in this study is to mount the LiDAR upward so that the top part of the dump truck can be observed. However, this also creates a trade-off because the blind area on the ground becomes larger and makes it harder to detect surrounding objects. Therefore, a comprehensive design discussion is required regarding sensor placement. Finally, parameters related to negative point clouds were tuned for specific scenarios and their adaptability to other cases was not validated. For future work, we intend to develop an automatic parameter searching approach to improve adaptability across datasets.

## 7. Conclusions

In this study, we proposed a two-stage method for pose estimation and size classification of dump trucks with unknown specifications. The proposed method performs pose estimation and size classification by NDT in parallel with multiple templates that represent different size categories. For appropriate size classification, we incorporate negative point clouds into the conventional NDT score. The performance of the proposed method was evaluated using data acquired in a real-world environment. The results demonstrated that the proposed method could estimate the pose of dump trucks robustly and classify their size by incorporating negative point clouds. Based on these results, the proposed method will contribute to not only wheel loader operation but also the overall automation of soil loading onto dump trucks at construction sites.

However, as described in Section 6, several limitations, such as the limited number of size categories, the evaluation under adverse environments, the investigation on LiDAR placement, and the requirement for more adaptable parameter settings, remain to be addressed. It is necessary for future work to address these issues to enhance the robustness and applicability of the proposed method in real construction environments.

## Figures and Tables

**Table 1:** Specifications of the Livox HAP LiDAR used in this evaluation [40].

Laser Wavelength	905 nm
Laser Safety	Class 1 (IEC 60825-1:2014 [41])
Detection Range (100 klx)	150 m @ 10% reflectivity
FOV	120° (Horizontal) × 25° (Vertical)
Distance Random Error (1σ @ 20 m)	<2 cm
Angular Random Error (1σ)	<0.1°
Beam Divergence	0.28° (Vertical) × 0.03° (Horizontal)
Angular Resolution @ ROI	0.23° (Vertical) × 0.18° (Horizontal)
Point Rate	452,000 points/s (first or strongest return)

**Table 2:** Evaluation score and error results for 12 settings.

Case	Orientation	Score	$\mathrm{Error}\;t_{x}$ [m]	$\mathrm{Error}\;t_{y}$ [m]	$\mathrm{Error}\;\phi_{z}$ [rad]
1-A	✓	0.94	0.01	0.10	−0.012
1-B	✓	0.93	0.08	0.11	−0.017
1-C	✓	1.09	0.10	0.16	0.006
1-D	✓	0.93	0.09	0.16	0.003
2-A	✓	1.06	−0.04	0.13	0.011
2-B	✓	0.94	−0.04	0.10	−0.004
2-C	✓	1.11	−0.04	0.11	0.019
2-D	×	0.94	-	-	-
3-A	✓	1.03	−0.07	0.12	0.015
3-B	✓	0.94	−0.02	0.13	0.006
3-C	×	0.78	-	-	-
3-D	✓	0.95	−0.03	0.08	0.006

$t_{x}$ and $t_{y}$ represent translational parameters along the *x*-axis and *y*-axis, respectively, and $\phi_{z}$ denotes the yaw angle. In “Orientation” column, “✓” denotes a correct estimation, while “×” denotes an incorrect estimation.

**Table 3:** Size categories of dump truck and their length and vessel height.

Size Category	Name	Length [m]	Vessel Height [m]
small	Small A	7.80	2.52
	Small B	7.86	2.21
medium	Medium	7.86	3.00
large	Large	8.32	3.10

**Table 4:** Confusion matrix of evaluation score before incorporating negative point cloud.

			Template			Size Classification
			Small A	Medium	Large	
Input	small	Small A	1.18	**1.21** *	1.02	×
		Small B	1.06	**1.33** *	1.15	×
	medium	Medium	1.03	**1.34** *	1.04	✓
	large	Large	1.04	1.11	**1.18** *	✓

Highest score for each observed point cloud is highlighted in bold and marked with an asterisk. In “Size Classification” column, “✓” denotes a correct estimation, while “×” denotes an incorrect estimation.

**Table 5:** Confusion matrix of evaluation score after incorporating negative point cloud.

			Template			Size Classification
			Small A	Medium	Large	
Input	small	Small A	1.17 *	0.93	0.52	✓
		Small B	1.00 *	0.79	0.52	✓
	medium	Medium	1.02	1.33 *	0.92	✓
	large	Large	1.04	1.11	1.14 *	✓

Highest score for each observed point cloud is highlighted in bold and marked with an asterisk. In “Size Classification” column, “✓” denotes a correct estimation.

**Table 6:** Computation time of proposed method when Small A dump truck is observed.

		Template		
		Small A	Medium	Large
Process	Preprocess	0.009 [s]	0.010 [s]	0.009 [s]
	NDT-based pose estimation	0.088 [s]	0.090 [s]	0.088 [s]
	Size classification with negative point clouds	0.034 [s]	0.032 [s]	0.026 [s]
	Total	0.131 [s]	0.132 [s]	0.123 [s]

## Data Availability

The original contributions presented in this study are included in the article.

## Abbreviations

The following abbreviations are used in this manuscript:

ICP	Iterative Closest Point
NDT	Normal Distribution Transform

## References

[1] Labour Force Survey; e-Stat Portal Site of Official Statics of Japan. https://www.e-stat.go.jp/en/stat-search/files?page=1&layout=datalist&toukei=00200531&tstat=000001226583&cycle=7&tclass1=000001226584&tclass2=000001226585&tclass3val=0.

[2] Dadhich S., Sandin F., Bodin U., Andersson U., Martinsson T. (2019). Field test of neural-network based automatic bucket-filling algorithm for wheel-loaders. Autom. Constr..

[3] Eriksson D., Ghabcheloo R., Geimer M. (2024). Optimizing bucket-filling strategies for wheel loaders inside a dream environment. Autom. Constr..

[4] Wang Y., Liu X., Ren Z., Yao Z., Tan X. (2024). Synchronized path planning and tracking for front and rear axles in articulated wheel loaders. Autom. Constr..

[5] Li Y., Dong W., Zheng T., Wang Y., Li X. (2025). Scene-Adaptive Loader Trajectory Planning and Tracking Control. Sensors.

[6] Cao B., Liu X., Chen W., Li H., Wang X. (2023). Intelligentization of wheel loader shoveling system based on multi-source data acquisition. Autom. Constr..

[7] Kumar M., Ekevid T., Löwe W. (2024). Operator model for wheel loader short-cycle loading handling. Autom. Constr..

[8] Biber P., Strasser W. The normal distributions transform: A new approach to laser scan matching. Proceedings of the 2003 IEEE/RSJ International Conference on Intelligent Robots and Systems (IROS 2003).

[9] Besl P.J., McKay N.D. (1992). A method for registration of 3-D shapes. IEEE Trans. Pattern Anal. Mach. Intell..

[10] Lee J.-H., Lee J., Park S.-Y. (2022). 3D pose recognition system of dump truck for autonomous excavator. Appl. Sci..

[11] Sugasawa Y., Chikushi S., Komatsu R., Louhi Kasahara J.Y., Pathak S., Yajima R., Hamasaki S., Nagatani K., Chiba T., Chayama K. (2021). Visualization of Dump Truck and Excavator in Bird’s-eye View by Fisheye Cameras and 3D Range Sensor. Intelligent Autonomous Systems 16.

[12] An Y., Xu H., Guo Y., Qian J., Sun Z., Xie L. Point cloud registration network based on dual-attention mechanism for truck pose estimation. Proceedings of the 2024 39th Youth Academic Annual Conference of Chinese Association of Automation (YAC).

[13] Zhang X., Xu W., Dong C., Dolan J.M. Efficient L-shape fitting for vehicle detection using laser scanners. Proceedings of the IEEE Intelligent Vehicles Symposium (IV).

[14] Liu Y., Liu B., Zhang H. Estimation of 2D bounding box orientation with convex-hull points—A quantitative evaluation on accuracy and efficiency. Proceedings of the 2020 IEEE Intelligent Vehicles Symposium (IV).

[15] Baeg J., Park J. Oriented bounding box detection robust to vehicle shape on road under real-time constraints. Proceedings of the 2023 IEEE 26th International Conference on Intelligent Transportation Systems (ITSC).

[16] Yan Y., Mao Y., Li B. (2018). SECOND: Sparsely Embedded Convolutional Detection. Sensors.

[17] Lang A.H., Vora S., Caesar H., Zhou L., Yang J., Beijbom O. PointPillars: Fast Encoders for Object Detection From Point Clouds. Proceedings of the IEEE/CVF Conference on Computer Vision and Pattern Recognition.

[18] Shi S., Guo C., Jiang L., Wang Z., Shi J., Wang X., Li H. PV-RCNN: Point-Voxel Feature Set Abstraction for 3D Object Detection. Proceedings of the IEEE/CVF Conference on Computer Vision and Pattern Recognition.

[19] Zhang T., Vosselman G., Elberink S.J.O. (2017). Vehicle recognition in aerial lidar point cloud based on dynamic time warping. IISPRS Ann. Photogramm. Remote Sens. Spat. Inf. Sci..

[20] Kraemer S., Stiller C., Bouzouraa M.E. Lidar-based object tracking and shape estimation using polylines and free-space information. Proceedings of the 2018 IEEE/RSJ International Conference on Intelligent Robots and Systems (IROS).

[21] Kraemer S., Bouzouraa M.E., Stiller C. Simultaneous tracking and shape estimation using a multi-layer laserscanner. Proceedings of the 2017 IEEE 20th Conference on Intelligent Transportation Systems (ITSC).

[22] Monica J., Chao W.-L., Campbell M. Sequential joint shape and pose estimation of vehicles with application to automatic amodal segmentation labeling. Proceedings of the 2022 IEEE International Conference on Robotics and Automation (ICRA).

[23] Ding W., Li S., Zhang G., Lei X., Qian H. Vehicle pose and shape estimation through multiple monocular vision. Proceedings of the 2018 IEEE International Conference on Robotics and Biomimetics (ROBIO).

[24] Monica J., Campbell M. Vision only 3-D shape estimation for autonomous driving. Proceedings of the IEEE/RSJ International Conference on Intelligent Robots and Systems (IROS).

[25] Magnusson M., Nuchter A., Lorken C., Lilienthal A.J., Hertzberg J. Evaluation of 3D registration reliability and speed—A comparison of ICP and NDT. Proceedings of the 2009 IEEE International Conference on Robotics and Automation (ICRA).

[26] Segal A.V., Haehnel D., Thrun S. Generalized-ICP. Proceedings of the Robotics: Science and Systems (RSS).

[27] Aoki Y., Goforth H., Srivatsan R.A., Lucey S. PointNetLK: Robust & Efficient Point Cloud Registration Using PointNet. Proceedings of the IEEE/CVF Conference on Computer Vision and Pattern Recognition.

[28] Charles R.Q., Su H., Kaichun M., Guibas L.J. PointNet: Deep Learning on Point Sets for 3D Classification and Segmentation. Proceedings of the IEEE/CVF Conference on Computer Vision and Pattern Recognition.

[29] Wang Y., Solomon J. Deep Closest Point: Learning Representations for Point Cloud Registration. Proceedings of the IEEE/CVF Conference on Computer Vision and Pattern Recognition.

[30] Wang Y., Sun Y., Liu Z., Sarma S.E., Bronstein M.M., Solomon J.M. (2019). Dynamic Graph CNN for Learning on Point Clouds. ACM Trans. Graph..

[31] Li Y., Niu T., Qin T., Yang L. Machine vision based autonomous loading perception for super-huge mining excavator. Proceedings of the 2021 IEEE 16th Conference on Industrial Electronics and Applications (ICIEA).

[32] Suzuki T., Ohno K., Kojima S., Miyamoto N., Suzuki T., Komatsu T., Nagatani K. (2021). Estimation of articulated angle in six-wheeled dump trucks using multiple GNSS receivers for autonomous driving. Adv. Robot..

[33] Stentz A., Bares J., Singh S., Rowe P. (1999). A Robotic Excavator for Autonomous Truck Loading. Auton. Robot..

[34] Phillips T.G., McAree P.R. (2018). An evidence-based approach to object pose estimation from LiDAR measurements in challenging environments. J. Field Robot..

[35] Wang M., Huang R., Xie W., Ma Z., Ma S. (2025). Compression Approaches for LiDAR Point Clouds and Beyond: A Survey. ACM Trans. Multimed. Comput. Commun..

[36] Wang M., Huang R., Liu Y., Li Y., Xie W. (2025). suLPCC: A Novel LiDAR Point Cloud Compression Framework for Scene Understanding Tasks. IEEE Trans. Ind. Inform..

[37] Biber P., Fleck S., Strasser W. (2004). A probabilistic framework for robust and accurate matching of point clouds. Lect. Notes Comput. Sci..

[38] Magnusson M. (2009). The three-dimensional normal-distributions transform: An efficient representation for registration, surface analysis, and loop detection. Örebro Stud. Technol..

[39] AMD Ryzen™ 7 7730U, AMD. https://www.amd.com/en/products/processors/laptop/ryzen/7000-series/amd-ryzen-7-7730u.html.

[40] Livox HAP (T1) User Manual, Livox. https://dl.djicdn.com/downloads/Livox/HAP/HAP(T1)_User_Manual_V1.2_EN.pdf.

[41] (2014). Safety of Laser Products-Part 1: Equipment Classification and Requirements.

